# Violent Recidivism: A Long-Time Follow-Up Study of Mentally Disordered Offenders

**DOI:** 10.1371/journal.pone.0025768

**Published:** 2011-10-11

**Authors:** Thomas Nilsson, Märta Wallinius, Christina Gustavson, Henrik Anckarsäter, Nóra Kerekes

**Affiliations:** 1 National Board of Forensic Medicine, Gothenburg, Sweden; 2 Forensic Psychiatry, Institute of Neuroscience and Physiology, University of Gothenburg, Gothenburg, Sweden; 3 Forensic Psychiatry, Department of Clinical Sciences, Lund University, Lund, Sweden; 4 Swedish Prison and Probation Service, R&D Unit, Gothenburg, Sweden; The University of Queensland, Australia

## Abstract

**Background:**

In this prospective study, mentally disordered perpetrators of severe violent and/or sexual crimes were followed through official registers for 59 (range 8 to 73) months. The relapse rate in criminality was assessed, compared between offenders sentenced to prison versus forensic psychiatric care, and the predictive ability of various risk factors (criminological, clinical, and of structured assessment instruments) was investigated.

**Method:**

One hundred perpetrators were consecutively assessed between 1998 and 2001 by a clinical battery of established instruments covering DSM-IV diagnoses, psychosocial background factors, and structured assessment instruments (HCR-20, PCL-R, and life-time aggression (LHA)). Follow-up data was collected from official registers for: (i) recidivistic crimes, (ii) crimes during ongoing sanction.

**Results:**

Twenty subjects relapsed in violent criminality during ongoing sanctions (n = 6) or after discharge/parole (n = 14). Individuals in forensic psychiatric care spent significantly more time at liberty after discharge compared to those in prison, but showed significantly fewer relapses. Criminological (age at first conviction), and clinical (conduct disorder and substance abuse/dependence) risk factors, as well as scores on structured assessment instruments, were moderately associated with violent recidivism. Logistic regression analyses showed that the predictive ability of criminological risk factors versus clinical risk factors combined with scores from assessment instruments was comparable, with each set of variables managing to correctly classify about 80% of all individuals, but the only predictors that remained significant in multiple models were criminological (age at first conviction, and a history of substance abuse among primary relatives).

**Conclusions:**

Only one in five relapsed into serious criminality, with significantly more relapses among subjects sentenced to prison as compared to forensic psychiatric care. Criminological risk factors tended to be the best predictors of violent relapses, while few synergies were seen when the risk factors were combined. Overall, the predictive validity of common risk factors for violent criminality was rather weak.

## Introduction

Violent crimes committed by mentally disordered offenders have received a great deal of media attention during later years, not least in Sweden, where a series of slayings in 2003, including the murder of the Foreign Minister, were committed by mentally ill perpetrators. These tragedies intensified the common notion that individuals with mental health problems are particularly dangerous and unpredictable. Believes of this kind have also been exploited in popular culture, and the “mad psychopathic killer” who may turn his rage upon any innocent bystander is a familiar figure to us all. But – what do the facts really tell us about mentally disordered offenders and their propensity to act violently?

During the 1990s, evidence indicating mental disorders as a cause of violence in the population accumulated rapidly, mainly in the form of data associating major mental disorders with violent criminality (e.g.: [1], [2], [3], [4]). In the large-scale MacArthur study of mental disorders and violence, Monahan and colleagues reported that 27.5% of their patients committed post-discharge violent acts during a one-year follow-up study period [5], but they could also show that there were no higher propensity for violence among psychiatric patients without concomitant substance abuse than among other residents in the same neighbourhood [5], [6]. Using population registers, Fazel and Grann found the population-attributable risk fraction of severe mental illness on violent criminality to be no more than 5% [7]. In a later study, Fazel and coworkers demonstrated that most of the risk for violent offending associated with schizophrenia and other psychoses was mediated by comorbid substance abuse, and that the risk of violent acts among the subjects with psychoses and substance abuse differed little from that among those with substance abuse but without psychoses [8]. When unaffected siblings were used as controls, the risk increase carried by the psychotic disorders was even less pronounced [8]. A considerable subgroup of patients with major mental disorders also had disruptive behaviour problems and substance abuse before developing psychosis [9]. As early-onset conduct disorder is a core risk factor for later criminality and entails susceptibility for mental disorders, the causal link between major mental disorder and violent criminality, a link that has long been presumed on the strength of a large body of high-quality reports, seems less convincing today. Instead, the risk increase for violent crime associated with major mental disorders seems to be confounded by psychosocial factors, substance abuse, and conduct disorder with onset before the development of psychosis [6], [8], [10].

Another question is whether mentally disordered offenders are more prone to re-offend than offenders without mental disorders. Though working with a variety of follow-up periods and cultural contexts as well as with different samples of mentally disordered offenders, international studies agree that the overall picture of violent recidivism among forensic psychiatric patients is surprisingly modest, varying between 6% and 15% [11], [12], [13]. This vulnerable group thus appears to be far less prone to relapse into violence than offenders sentenced to prison, among whom more than one in three are reconvicted [14], [15].

The notion that mentally disordered individuals are dangerous as they are more prone to relapse in violent behaviour has also prompted researchers to develop risk assessment tools aimed at identifying the individuals most liable to violent recidivism. A broad array of criminological (such as criminal history and personal demographics) and clinical risk factors (such as diagnoses of mental disorders, including antisocial personality disorder) has been studied, and great efforts have gone into the development of risk assessment tools, such as the Violence Risk Appraisal Guide (VRAG) [16], [17] and the Historical, Clinical, and Risk Management 20-item scale (HCR-20) [18], facilitating actuarial (i.e. statistical) approaches to clinical and criminological risk factors alike.

Bonta and co-workers showed already in 1998 [19] that criminal history variables outperformed clinical risk variables in the prediction of violent recidivism. The role of criminological versus clinical risk factors in predicting violence has since then also been studied in the MacArthur study of mental disorder and violence [5], showing that the different risk factors had complex relations to the outcome variable (violence), and that the factors involved could play either a protective or a predictive role depending on patient characteristics (e.g. ethnical background, family circumstances). Based on these results, Monahan and co-workers argued for an “interactional tree” approach to violence risk assessment, the so called Classification of Violence Risk (COVR) instrument [5], [20], but no evidence for the superiority of this instrument as compared to other clinical and actuarial instruments has been presented so far.

Besides the specialized risk assessment instruments, high scores on the Psychopathy Checklist (Revised) (PCL-R) [21], [22], originally created to study psychopathic personality traits, have in many studies from various countries been associated with violent recidivism [23], [24]. However, in a recently published study [25], the criminal history variables in the Antisocial Facet 4 of the PCL-R fell out as the only true predictor of criminal recidivism, without any incremental effects from the other three Facets (Interpersonal, Affective, and Lifestyle).

A circumstance contributing to the uncertainty about the usefulness of different clinical predictors in risk assessments for violence is that study samples have been recruited from four different types of populations: the general population, discharged psychiatric patients, mentally disordered offenders, and offenders in general. Psychotic disorders in the general population have, for example, been associated with an increased risk of violent crime as compared to the extremely low risk in the non-sentenced general population [7] even after correction for socio-demographic confounders and comorbid substance abuse [26], [27], whereas psychotic disorder in discharged patient samples has emerged as a protective factor in relation to the relatively high base-rate of recidivism in this group [19].

In a review comparing different clinical and actuarial measures for violence risk prediction, Dolan and Doyle [28] found that systematic and structured approaches enhanced the clinical prediction of violent outcomes with the PCL-R as a key predictor. Compilation of their result clearly shows, however, that the differences between the studied instruments (VRAG, HCR-20, and PCL-R) are rather small, with areas under Receiver Operating Characteristics (ROC) curves (AUC) ranging between 0.70 and 0.80 for all studied instruments, indicating modest predictive ability. A recently presented meta-analysis based on 68 original studies covering nine of the most commonly used risk assessment instruments (with HCR-20, PCL-R, and VRAG included among others) did not alter the picture, since none of the studied instruments showed a median AUC above 0.78 [29]. To our knowledge, no study with acceptable methodology has shown an AUC clearly above 0.80 (i.e. in the good prediction range) for any risk assessment method. Complex risk assessment tools, such as the COVR [5] and the HCR-20 [18], and actuarial risk assessment instruments, such as the VRAG [17] and the Offender Group Reconviction Scale (OGRS) [30], have all so far been shown to have at best modest predictive ability. In a survey of the research about risk assessment of mentally disordered individuals [31], it was concluded that the finest available methods at most could identify three male dangerous patients out of four (75%), while there was no evidence-base at all for risk assessments of females and ethnic minorities.

All in all, mentally disordered offenders seem to be ascribed an exaggerated propensity to reoffend, and the knowledge about criminological as well as clinical risk factors is confused by data from different types of samples (e.g. discharged psychiatric patients, and mentally disordered offenders). The aims of the present study are (i) to describe the relapse rate in violent crimes (reconvictions) in mentally disordered violent offenders and compare these findings between the subjects sentenced to compulsory forensic psychiatric treatment versus those sentenced to prison, and (ii) to test the predictive ability of common criminological and clinical risk factors as well as structured assessment instruments for violence risk and for aggression in a truly prospective, long-time, clinical follow-up study of mentally disordered violent offenders sentenced to prison or to compulsory forensic psychiatric treatment.

## Methods

### Study subjects

One hundred consecutively recruited violent and/or sexual offenders (92 men and 8 women, 17–76 years old, median age 30 years) were prospectively included in this study between 1998 and 2001. All had been charged with severe violent and/or sexual crimes and court-referred to the Department of Forensic Psychiatry in Gothenburg for pre-trial forensic psychiatric investigations. At the subsequent trials, all were found guilty and sentenced: 46 (18–76 years old, median age 30 years) to compulsory forensic psychiatric care and 54 (17–68 years old, median age 32 years) to prison. Baseline data from this study, mainly on neuropsychiatric and biological covariates to violent criminality and aggression, has previously been reported [32], [33], [34], [35], including detailed descriptions of the participants.

### Procedures

The study plan was approved by the Research Ethics Committee at the University of Gothenburg, including the collection of follow-up data. On the Committees recommendation potential study subjects were given both oral and written information about the study, and included after they had given their oral consent to participate in it. This procedure was in accordance with the existing law at that time. An individual written research protocol was then created for each participating subject, also stating that they had approved to take part in the research project. Potential Baseline data covering a broad array of psychiatric, psychological, and psychosocial variables was collected during the forensic psychiatric investigations. This procedure has been described in detail in a previous publication [35]. Briefly, study subjects were included by criteria defining the type of severe violent and sexual crimes. For study purposes, the initial legal classification of the crime(s) was used as long as the description of the criminal act remained unchanged throughout the judicial process.

### Measures

#### (i) Demographic and criminological data

Data covering historical, demographic, and criminological aspects including age at first conviction, number of previous convictions for aggravated violence, number of prison convictions, number of previous crimes (no crime, one crime, two to four, and ≥five crimes), time-span between previous crimes (no crime, >five years, two to five years, and <two years between previous crimes), sexual abuse during childhood, substance abuse problems and criminality among caregivers during childhood, and otherwise aggravating circumstances during childhood were collected by means of a structured research protocol using records available for the forensic psychiatric investigations in addition to interviews.

#### (ii) Structured assessment instruments of violence risk and aggression

The Historical, Clinical, Risk Management-20 (HCR-20) [18] was rated as a measure of risk for violent criminal recidivism. The HCR-20 is a 20-item risk assessment checklist developed for the purpose of assisting the structured clinical judgment in violence risk assessments. The items are rated on a three-point scale, from “not present” to “definitely present”. For the current study, only the 15 historical and clinical items of the HCR-20 were rated and used as a total score, since the risk management items could not be rated due to their focus on individual treatment and management plans that would not be implemented until after the court had pronounced the verdict and it had gained legal force.

The Psychopathy Checklist-Revised (PCL-R) [21], [22] was used for assessment of psychopathic personality traits. The PCL-R is a 20-item rating scale with items rated on a three-point scale (0 = does not apply, 1 = may apply or in some respects applies, 2 = does apply) measuring the interpersonal, emotional, and behavioural aspects of the construct of psychopathy. The PCL-R ratings were performed by an especially trained forensic social investigator on the basis of the assessments made during the forensic psychiatric investigations and on extensive file and register reviews in each case. The PCL-R was treated as a unified construct by its total score.

The Life History of Aggression (LHA) [36] was used as a measure of aggressive behaviour. The LHA was originally developed within research on neurobiological correlates to aggression in order to measure the frequency of 11 different aggressive behaviours. The items are rated on a five-point scale based on the number of occurrences of the behaviour since adolescence, from 0 (“no events”) to 5 (“so many events that they cannot be counted”). Three subscales have been created: (i) aggression with items measuring temper tantrums, physical fights, verbal aggression, physical assaults on people or animals, and assaults on property, (ii) self-directed aggression that includes items regarding self-injurious behaviour and suicide attempts, and the (iii) consequences/antisocial behaviour subscale depicting school disciplinary problems, problems with supervisors at work, and antisocial behaviour with or without police involvement. In the present study, the LHA was first administered as a self-rating instrument followed by careful reviews of these reports in relation to the forensic psychiatric investigation and all available records and file reports for each participant. The total scale score based on an average of the self-rated and expert-rated assessments was used in the analyses.

#### (iii) Clinical assessments

Psychiatric diagnoses for Axes I and II according to the Diagnostic and Statistical Manual of Mental Disorders 4^th^ edition (DSM-IV) [37] were determined on the basis of the Structured Clinical Interview for the Axis I and II (SCID I and II) [38], [39] applied by expert assessors with access to the extensive information obtained during the forensic psychiatric investigations.

The diagnosis of conduct disorder (CD) was primarily assigned based on the SCID-II, and, when possible, on a semi-structured collateral interview on childhood neurodevelopment with a relative who had known the participant as a child.

#### (iv) Follow-up data

Follow-up data for the following outcomes: (i) recidivistic crimes (all types, particularly sexual and violent crimes), defined as reconvictions, was during 2005 obtained from registers of the National Council for Crime Prevention, the National Prison and Probation Administration, and the Central Archives of the National Board of Forensic Medicine, (ii) violent recidivism during sanction (forensic psychiatric involuntary treatment/prison) was also notified. Violent recidivism was defined as all violence-related convictions, such as murder, assault (also aggravated), intimate partner violence, robbery, arson, exposing somebody to danger, and violations of the legislation against carrying arms/knives in public places. Additional information about mortality and causes of death was collected from registers provided by the National Board of Health and Welfare. Follow-up time included the period from inclusion in the study until the first of January 2005 unless death occurred earlier.

### Statistical analyses

All analyses were conducted with the PASW 18.0 software, using two-tailed p-values. As the data could not be assumed to be normally distributed, non-parametric statistics were consistently used. Between-group differences were examined using Fischer's exact tests, and relations between continuous variables were analyzed by Spearman's rank-order correlations. A Kaplan-Meier survival analysis with a Log-Rank test was used to compare time in months until violent relapse for the two different forms of sanction, i.e. prison and forensic psychiatric care. ROC-analyses were performed to examine the predictive ability of the different risk factors (e.g. age at first conviction, number of convictions for aggravated violence, number of convictions, PCL-R total score, LHA total score, and HCR-20 total score on historical and clinical items) for criminal recidivism (reconviction). The ROC-curves was also used to identify the optimal inflection point for the single risk factors, i.e. the cut-off on the continuous scale where the trade–off between sensitivity and specificity reached its peak. Finally, binary logistic regression analyses were performed for the criminological risk factors and for the combined set of clinical risk factors and assessments from structured instruments with relapse in violent criminality as dependent variable. The predictive classifications that emerged from the optimal inflection point of the different ROC-curves, and from the regression models, were also used to calculate sensitivity, specificity, and positive predictive value (PPV) as well as negative predictive value (NPV) for both single and combined sets of risk factors. All analyses presented in the results section were performed for the total study group with one missing case due to mismatch of social security codes (N = 99), but since we also lacked baseline data on some risk factors in some cases, the number of subjects varies between 99 and 87 in the different analyses.

## Results

### Outcome at follow-up

Basic data for the entire follow-up period, for time spent in sanctions as well as for time at liberty/risk, together with the number of cases with a violent reconviction or any reconviction for criminality during the follow-up period is presented in Table 1, for the total group as well as for the two groups sentenced either to compulsory forensic psychiatric treatment or prison. The follow-up period included almost five years (with a minimum of 8 and a maximum of 73 months), showing a small but significant difference between the subjects in forensic psychiatric care (61 months) and those in prison (58 months). The shortest follow-up periods were due to deaths (three suicides after 8, 22, and 32 months, respectively). All three deaths occurred in the forensic psychiatric care group, and one of them (with a follow-up period of 22 months) had relapsed into a violent crime shortly before taking his life.

**Table 1:** Basic descriptions concerning aspects of the follow-up period (months), violent criminal relapses, all criminal relapses, and psychiatric diagnoses at baseline for the total group, those in forensic psychiatric care, and those sentenced to prison.

*Follow-up data, number of relapses, and clinical characteristics*	*Total group (n = 99)*	*Forensic psychiatric care (n = 46)*	*Prison group (n = 53)*	*p-value*
Follow-up period (mean (SD), min - max)	59.3 (±10.9) 8–73	61.3 (±11.1) 8–73	57.5 (±10.4) 22–73	0.025
Time in psychiatric treatment or prison including conditional release (mean (SD), min - max)	28 (±19.8) 0–73	17.5 (±17.7) 0–63	37 (±16.9) 1–73	<0.001
Time spent at liberty after discharge/time at risk (mean (SD), min - max)	30.9 (±22.4) 0–72	43.3 (±19.7) 0–72	20.1 (±18.7) 0–60	<0.001
Subjects in treatment/prison at the end of the follow-up period; n (%)	10 (10%)	1 (2%)	9 (17%)	0.018
Subjects on long-term leave/parole at the end of the follow-up period; n (%)	7 (7%)	0	7 (13%)	0.014
Total number of relapses in violent criminality*; n (%)	20 (20%)	5 (11%)	15 (28%)	0.044
Total number of relapses in criminality**; n (%)	27 (27%)	7 (15%)	20 (38%)	0.014
Relapses in violent criminality during ongoing forensic psychiatric treatment/prison sanction; n (%)	6 (6%)	2(4%)	4 (8%)	ns
Relapses in violent criminality during conditional release/after discharge; n (%)	14 (14%)	3 (7%)	11 (21%)	0.05
DSM-IV axis I diagnosis of psychosis at baseline; n (%)	20 (20%)	15 (33%)	5 (9%)	0.006
At least one DSM-IV axis II PD diagnosis; n (%)	66 (67%)	29 (63%)	37 (70%)	ns
DSM-IV axis II diagnosis of APD; n (%)	42 (42%)	18 (39%)	24 (45%)	ns
DSM-IV diagnosis of substance abuse/dependence; n (%)	52 (53%)	23 (50%)	29 (55%)	ns
DSM-IV diagnosis of CD during childhood; n (%)	47 (48%)	19 (41%)	28 (53%)	ns

*Consisting of all violent reconvictions during the follow-up period (e.g. one murder, one arson, one case of exposing somebody to danger, two aggravated assaults, five assaults, three aggravated unlawful thefts/robberies, two cases of intimate partnership violence, and five violations of the legislation against carrying arms/knives in public places.

**Consisting of all reconvictions during the follow-up period (e.g. besides violent criminality also drug crimes, shoplifting, and traffic offences).

In general, the time spent in prison was twice as long as the time spent in forensic psychiatric care, and the subjects sentenced to psychiatric treatment spent significantly more time (on average 43 months) at liberty/risk than the prisoners (on average 20 months). Nevertheless, reconvictions for violent criminality were significantly more common among those sentenced to prison as compared to those sentenced to forensic psychiatric care. Totally, 20% (20 subjects) of the entire study population relapsed once into violent criminality during the follow-up period, demonstrating about four violent relapses per 100 patient-years. When all types of criminality were taken into account, another 7% (7 subjects, 5 in the prison group and 2 in the forensic care group) were reconvicted (3 for drug crimes, 1 for shoplifting, and 3 for traffic offences).

Clinical characteristics (number of individuals at baseline with DSM-IV psychotic disorders, at least one Axis II personality disorder, antisocial personality disorder, substance abuse/dependence, and/or childhood CD) are also presented in Table 1. The only diagnostic difference between the two sanction groups was that psychotic disorders were significantly more common among those sentenced to forensic psychiatric treatment (as expected in view of the legal criteria for such a sentence).

**Figure 1:**
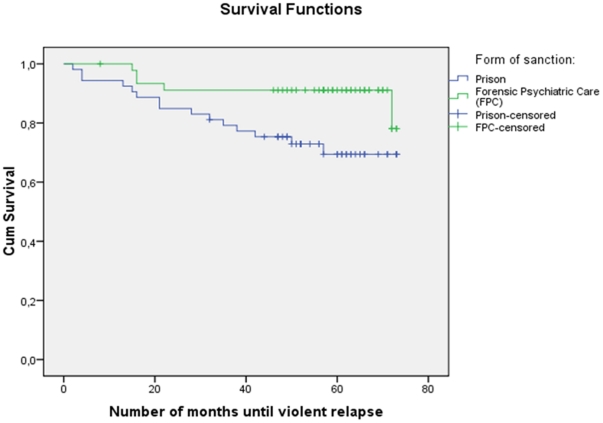
Kaplan-Meier survival analysis comparing time in months until violent relapse for the two sanctions prison and forensic psychiatric care (p<0.024). (The mark of censored data indicates the end of an individual follow-up period.)

Time (in months) until first violent relapse (reconviction) was compared between the two sanction groups by a Kaplan-Meier survival analysis (Figure 1), which also took censored data into account (i.e. differences in duration of the individual follow-up period), showing that the relapses were fewer and occurred at the beginning of the follow-up period among those sentenced to forensic psychiatric treatment, whereas the pattern of incidents among those sentenced to prison displayed relapses spread out over the entire follow-up period. The difference between the two groups was statistically significant (p<0.024).

### Criminological and clinical risk factors

Baseline criminological risk variables and structured assessment instruments aiming to predict recidivism in violent criminality (reconvictions) showed associations with the outcome as detailed in Table 2 for the continuous variables (Spearman correlation coefficients). The correlations were overall small to modest, not exceeding 0.33, as found for two assessment instruments: total scores on the PCL-R and the LHA. Among the criminological risk factors, “age at first conviction” showed the strongest, though small, association with violent recidivism followed by “number of prison convictions”.

**Table 2:** Correlation coefficients (Spearman's rho) between criminological risk factors and scores on structured assessment instruments in relation to violent recidivism during the follow-up period.

Criminological risk factors	Violent recidivism(n)
Age at first conviction	−0.28**(99)
Number of convictions for aggravated violence	0.18(95)
Number of prison convictions	0.21*(96)
**Structured assessment instruments**	
PCL-R total score	0.33**(99)
HCR-20, total score on historical and clinical part	0.29**(96)
LHA total score	0.33**(91)

*Correlation is significant at the 0.05 level (2-tailed).

**Correlation is significant at the 0.01 level (2-tailed).

Associations between clinical risk factors and violent recidivism are shown in Table 3. Among the clinical risk factors, a diagnosis of CD during childhood fell out as significant (p = 0.002), occurring in a majority of those who actually relapsed (80% of recidivists fulfilled criteria for childhood CD as compared to 39% of non-recidivists), while the only other significant risk factor was the diagnosis substance abuse/dependence (p = 0.027).

**Table 3:** Clinical risk factors and violent recidivism during the follow-up period.

Clinical risk factors	No recidivism(n = 79)	Violent recidivism(n = 20)	P(Fisher's Exact test)
Psychosis*:			
Yes	15 (19%)	5 (25%)	
No	64 (81%)	15 (75%)	ns
Substance abuse/dependence:			
Yes	37 (47%)	15 (75%)	
No	42 (53%)	5 (25%)	0.027
Antisocial personality disorder:			
Yes	30 (38%)	12 (60%)	
No	49 (62%)	8 (40%)	ns
Conduct disorder:			
Yes	31 (39%)	16 (80%)	
No	48 (61%)	4 (20%)	0.002

*All diagnoses in the table are based on the DSM-IV.

Criminological risk factors covering individual aspects such as “number of” or “time-span between” previous crimes as well as childhood experiences and environmental circumstances during childhood/adolescence were assessed and compared between groups of recidivistic and non-recidivistic offenders. While the proportion of offenders with ≥5 previous crimes and the proportion of offenders with <2 years between earlier crimes were twice as large within the group of recidivists than among the non-recidivists, none of these risk factors was significantly related to violent recidivism (due to the small number of recidivists). Neither did any of the more environmental criminological risk factors, such as those reflecting unstable and insecure circumstances and histories of sexual abuse during childhood and/or adolescence, or primary relatives with substance abuse/dependence and/or criminality, show any statistically significant relation with violent recidivism.

To illustrate how the three continuous criminological risk factors (age at first conviction, number of convictions for aggravated violence, and number of prison convictions) together with the three assessment instruments (PCL-R total score, LHA total score, and HCR-20 total score) predicted violent criminal recidivism, ROC curves were plotted for the total group. ROC analyses showed modest predictive abilities for the criminological risk factor age at first conviction with an AUC of 0.70 (CI (95%) = 0.58–0.83, p = 0.005), and for all three assessment instruments, with an AUC of 0.71 (0.60–0.83, p = 0.004) for HCR-20, an AUC of 0.74 (0.62–0.86, p = 0.001) for PCL-R, and an AUC of 0.74 (0.62–0.86, p = 0.002) for LHA. Two of the criminological risk factors did not predict violent recidivism significantly; number of previous convictions for aggravated violence and number of prison convictions. The optimal inflection point for the criminological risk factor age at first conviction was 19 years, where an age ≤19 correctly classified 64% of all subjects with a sensitivity of 0.65 and a specificity of 0.63. The corresponding PPV and NPV was 0.31 and 0.88, respectively. For the assessment instrument HCR-20, the optimal inflection point was 12.5, where a score above that value correctly classified 63% of the studied subjects with a sensitivity of 0.80, a specificity of 0.57, a PPV of 0.32, and a NPV of 0.92. When it comes to PCL-R, the optimal inflection point was 11.5, where a score higher than that value correctly classified 70% of all subjects. In this case the corresponding value for the sensitivity of the prediction was 0.65, and for the specificity 0.71, with a PPV of 0.36 and a NPV of 0.89. LHA, finally, showed an optimal inflection point of 27, where a score above that value correctly classified 65% of all subjects with a sensitivity and a specificity of 0.65 in both cases, a PPV of 0.32, and a NPV of 0.88.

### Overall prediction of violent recidivism

Logistic regression analyses were used to identify the best criminological or clinical/structured assessment risk predictors for recidivism into violent criminality as the dependent variable (Table 4). The overall multiple regression model for the *criminological risk* factors correctly classified 85% of all subjects regarding violent recidivism, with a sensitivity of 0.41 and a specificity of 0.97. The PPV was 0.77 and the NPV 0.86. Two criminological risk factors were significant in the model: age at first conviction and substance abuse/dependence among primary relatives. The other regression model, using a combined set of *clinical/structured assessment risk* factors/scores as dependent variables, correctly classified 81% of all subjects with a sensitivity of 0.18 and a specificity of 0.96. In this model, the PPV was 0.50 while the NPV was 0.83. Neither the clinical risk factors nor the structured assessment scores in the regression model showed a significant association with violent recidivism.

**Table 4:** Binary logistic regression analyses for criminological risk factors, and for a combined set of clinical risk factors and structured assessment instruments, with recidivism into violent criminality as dependent variable.

Criminological risk factors	Violent criminality		
	Wald	Exp(β) (95% CI)	p
Age at first conviction	5.45	0.86 (0.76–0.98)	0.02
Number of convictions for aggravated violence	0.003	1.03 (0.35–3.06)	ns
Number of prison convictions	0.92	1.13 (0.88–1.44)	ns
Values based on number of previous crimes	0.05	0.88 (0.29–2.71)	ns
Values based on time between previous crimes	0.07	0.88 (0.33–2.34)	ns
Substance abuse/dependence among primary relatives	4.21	2.68 (1.05–6.86)	0.04
Criminality among primary relatives	3.37	0.11 (0.01–1.16)	ns
Unstable and insecure circumstances during childhood	0.01	0.94 (0.34–2.73)	ns
Sexually abused during childhood/adolescence	0.67	0.64 (0.22–1.86)	ns

Clinical risk factors and assessment instruments	Violent criminality		
	Wald	Exp(β) (95% CI)	p
DSM-IV diagnosis of psychosis	0.22	1.45 (0.31–6.76)	ns
DSM-IV diagnosis of substance abuse/dependence	0.48	1.71 (0.37–7.88)	ns
DSM-IV diagnosis of APD	1.14	0.41 (0.08–2.11)	ns
DSM-IV diagnosis of CD	2.89	5.91 (0.76–45.74)	ns
PCL-R total score	2.46	0.91 (0.80–1.03)	ns
HCR-20, total score on historical and clinical part	0.28	1.05 (0.87–1.27)	ns
LHA total score	0.44	0.98 (0.91–1.05)	ns

## Discussion

In this prospective long-time follow-up study, mentally disordered offenders who had committed severe violent and/or sexual crimes were followed for an average period of almost five years to determine the rate of violent recidivism and to quantify associations between criminological and clinical risk factors and reconvictions. Despite the long follow-up period, only 20 individuals (20%) were reconvicted for violent or violence-related crimes during the total follow-up period, resulting in a total reconviction rate of 27% when non-violent crimes were included. This recidivism rate is in line with the results from other long-term follow-up studies of mentally disordered offenders and patients discharged from special hospitals [11], [12], [13], [40].

The offenders were sentenced to either of two forms of sanctions: compulsory forensic psychiatric treatment or prison. When reconviction rates, regardless of whether the relapses occurred during ongoing sanction or after release, were compared between sanctions, a significant difference was seen in favour of the subjects in psychiatric care, who had only five (11%) reconvictions, while those sentenced to prison had 15 (28%). Judging from these results, and keeping in mind that offenders are not randomized to these sanctions but sorted by a detailed legal frame-work, risk management and prevention of criminal recidivism seems to work better in the forensic psychiatric care system than in the prison-parole system. A possible explanation for this divergence is that forensic psychiatric care – in contrast to prison – may be equipped to meet more individual, need-specific conditions. Another possible explanation is that the forensic psychiatric after-care programs may provide more adequate support, for example in the form of transitional accommodations. Also, the relapse patterns differed significantly between our two sanction groups, with relapses mainly in the beginning of the follow-up period in the forensic psychiatric group, which is in line with previous studies of discharged psychiatric patients [5], while the relapses were spread over the whole follow-up period in the prison group. A speculative explanation is that forensic psychiatric care may provide a continuous relationship between patient and staff leading to a stable treatment alliance which may deepen with time and thus prevent patients from relapsing, while the prison environment is disruptive, with sudden transfers and a distance between the prisoner and the prison staff.

A surprising finding was that more than one fourth of the violent recidivism generated by our whole study group occurred during ongoing forensic psychiatric treatment or term in prison. Some of the most serious violent crimes (e.g. one murder, one assault, and one case of intimate partnership violence) were in fact committed during ongoing sanctions. Even if it is important to consider “time at risk”, i.e. time spent at liberty after release, these findings also emphasize the importance of taking the sanction period into consideration with regard to preventive measures by focusing more on individual risk factors and risk management instead of solely concentrate on psychiatric diagnoses or on perimeter security.

The clinical assessments of mental disorders, personality disorders, substance abuse/dependence, and CD, showed similar characteristics in the two sanction groups with exception only for psychotic disorder, which by definition was significantly higher in the forensic psychiatric group as compared to the prison group. This agrees with the hypothesis that a diagnosis of psychosis may constitute a protective factor as the presence of a mental disorder is associated with less recidivism [19].

Univariate associations between clinical and criminological risk factors and violent reconvictions were also studied. Our results support previous findings that some criminological risk factors (such as age at first conviction and number of prison convictions) [19] are modestly associated with reconviction. However, in contrast to, for example, the MacArthur study [5], other criminological risk factors (such as the number of earlier crimes, substance abuse/dependence, and/or criminality among primary relatives) showed no significant relation to reconviction in our study population, while the structured assessment score of aggression was significantly associated with violent recidivism in both studies. This emphasizes the need for clinicians to use patients' history of aggression as a factor to consider when estimating the risk for future violent recidivism. Among the other clinical risk factors, scores on structured assessment instruments (HCR-20 and PCL-R) showed only modest relations with reconviction in analogy with previous studies reporting sensitivity, specificity, and/or predictive values that throw doubt on the widespread use of these methods today. Moreover, CD emerged as a significant clinical risk factor, lending support to the notion of a developmental path with childhood-onset disruptive behavioural problems evolving into life-course-persistent antisocial and aggressive behaviour with early-onset poly-drug abuse in a considerable number of affected individuals [41], [42].

Continuous criminological risk factors and structured assessment instruments were also tested by ROC analyses with regard to their ability to predict violent reconvictions. Scores on the assessment instruments were comparable to those found for the criminological variable age at first conviction. These results are quite modest and far from impressive, and it is thought-provoking that such a simple criminological variable as age at first conviction should posses almost the same predictive ability as elaborated assessment tools (HCR-20 or PCL-R) commonly used in risk assessment settings.

The ability within the two sets of risk factors, i.e. the criminological and the clinical combined with the structured assessment instruments, to overall predict violent reconviction was tested in separate regression analyses, and both sets of variables were found to possess about the same predictive ability; it was, however, only among the criminological variables that any significant predictors were found, namely age at first conviction and substance abuse/dependence among primary relatives. Once again, the historical variable of age at first conviction demonstrated its strong association with violent recidivism, just as Bonta and colleagues [19] showed in their meta-analysis. It was, on the other hand, rather surprising that none of the clinical risk factors possessed strong predictive ability. Since they all seemed to be too homogeneous, too one-dimensional, and without ability to add any incremental information to each other, it is near at hand to describe them as the same body in different disguises or as epiphenomenal “markers” for the common behavioural constellation described above.

The overall outcome of the two logistic regression models was, despite the fact that about 80% of the subjects were correctly classified, unsatisfactory, since the number of false positives was remarkably high. The clinical model could correctly predict about two out of four, while the criminological model could at best correctly predict about three out of four violent reconvictions. This is in line with what the Swedish Council on Technology Assessment in Health Care [31] stated in their survey of the field of risk assessment research, and this is probably the best we can get in predictions of future behaviour due to the variability that characterizes human behaviour. Since mentally disordered offenders constitute no exception from this condition, it might be high time to turn our efforts from identifying risk predictors to the development of treatment programs and post-discharge supportive utilities. A first step in that direction would be to identify the working components within forensic psychiatric care and post-discharge follow-up, since this treatment form seems to outperform traditional correctional treatment such as prison.

### Clinical implications

Clinicians should be aware that the rates of violent recidivism in offenders with mental disorders, contrary to popular opinion, tend to be rather modest, especially among mentally disordered offenders subjected to compulsory forensic psychiatric care. Caution is also required regarding the assessment of dangerousness, since it must be taken into consideration that the numbers of false positives often exceed the true positives. Instead of utilising complex and elaborated risk assessment tools, the same results can be achieved by looking at ordinary criminological facts such as the history of substance abuse and criminality, age, and gender.

### Limitations

There are, of course, limitations to the current study. First of all, the sample is rather small, thus limiting the ability to detect, for example, statistically significant associations between risk factors and outcome. This is especially relevant regarding the logistic regression analyses, which consists of several variables with varied distribution and an uncertain co-linearity that may give rise to random relations between predictors and outcome. Thus, the results of the logistic regression analyses must be interpreted with caution. The sample is also quite heterogeneous, with ages spanning from 17 to 76 years, and with 92 males and 8 females. On the other hand, this sample is collected within the regular clinical praxis, thus reflecting the real variability among mentally disordered offenders. Another limitation lies in the reliance on register information for the outcome variables. According to Monahan et al., [5] register-based information about violent criminality truly underestimates the prevalence of violence and antisocial behaviour. However, since this limitation applies with equal strength to each of the two groups with different sanctions, its impact on the comparison between these groups is negligible. All in all, generalizations from these results should be made with care and restricted to the type of population studied.

### Conclusion

To summarize, in this clinical sample of mentally disordered offenders, the rate of reconvictions over a five-year follow-up period amounted to 20%. The reconviction rates varied significantly with sanction form, i.e. the reconviction rate in the group with compulsory forensic psychiatric treatment compared to the prison group was one to three. Rather simple criminological risk factors, such as age at first conviction, were also as effective as structured risk assessment tools for predicting reconviction, while a set of criminological risk factors in a logistic regression analysis could correctly classify three out of four cases of reconviction compared to two out of four in a set of clinical risk factors combined with structured assessment instruments. The studied risk factors' overall rather poor ability to identify the individuals who relapsed into violent criminality provide a pertinent illustration of Rose's [43], [44] central argument, that risk factors are probabilistic concepts referring to a group of individuals and not to a specific individual, i.e. factors behind causes of incidence and not factors behind causes of cases. Another principal objection against risk assessment raised by our results concerns the relapse rate, which in this forensic psychiatric group was so low that predictions will be virtually meaningless. Every type of risk assessment requires a considerable incidence and the less common the predicted outcome, the more precarious the assessment, no matter how impressive the sensitivity and specificity of the method applied. Risk factors are thus not very well suited to screen for outcome such as violent recidivism on the individual level, since they are characterized by a rather poor discriminatory accuracy. Individual risk assessment, especially within the psychiatric field, should thus be carried out with great caution and be based on thorough knowledge about the individual case in question, and all who practice in this field should openly account for the lack of precision in their written or oral statements.
